# Asian American and Pacific Islander Access to Abortion During COVID-19: A Complex Interplay of Factors

**DOI:** 10.1089/heq.2022.0036

**Published:** 2022-08-24

**Authors:** Sruthi Chandrasekaran, Sung Yeon Choimorrow

**Affiliations:** ^1^ Ibis Reproductive Health, Cambridge, Massachusetts, USA.; ^2^ National Asian Pacific American Women's Forum, Washington, District of Columbia, USA.

**Keywords:** AAPI, COVID-19, abortion

## Abstract

Often stereotyped as the “model minority,” health care providers, lawmakers, and the general public regularly underestimate and ignore the health concerns of Asian American and Pacific Islanders (AAPIs). The COVID-19 pandemic has exposed the racism that AAPIs face—many communities report facing severe xenophobia during the pandemic, which has increased hesitancy to obtain needed medical treatment and heightened mental health issues at a time of isolation. The pandemic is also exacerbating the barriers that already exist in accessing abortion care—with travel restrictions, quarantine measures, lesser availability of appointments, and more burden on the health care staff and system. There has been no discussion on the impact of the pandemic on AAPIs' access to abortion care. We discuss challenges that are specific to AAPIs in accessing sexual and reproductive care, especially abortion, and how these are compounded by the lack of appropriate data and methods. We then discuss the added complexity that comes with accessing abortion care during a pandemic and provide recommendations for research methods to better reach these populations.

Often stereotyped as the “model minority,” health care providers, lawmakers, and the general public regularly underestimate and ignore the health concerns of Asian American and Pacific Islanders (AAPIs).^1^ The AAPI community is extremely diverse—its members represent over 30 countries, 20 distinct ethnic groups, and speak more than 100 languages.^2,3^ Of the 163 million women in the country, 10.7 million—or 6.5%—are AAPIs, and over 50% of all AAPI women are of reproductive age.^4^

The COVID-19 pandemic has exposed the racism that AAPIs face—many communities report facing severe xenophobia during the pandemic, which has increased hesitancy to obtain needed medical treatment and heightened mental health issues at a time of isolation.^5^ A study conducted by the National Asian Pacific American Women's Forum showed that 74% of AAPI women reported personally experiencing racism and/or discrimination in the past 12 months (i.e., 2021).^6^

Additionally, recent research indicates that AAPIs face increased disease severity and mortality rates from COVID-19.^5^ This situation is further exacerbated by additional factors such as AAPIs being a large part of the essential workforce and thereby being more likely to be exposed to the virus, and fear of experiencing violence and racism that may deter them from seeking care.^5^

The pandemic is also exacerbating the barriers that already exist in accessing abortion care—with travel restrictions, quarantine measures, lesser availability of appointments, and more burden on the health care staff and system.^7^ Barriers in terms of travel restrictions, quarantine measures, additional caregiving responsibilities, changes in economic stability, concerns around family's health, and fewer providers and staff available in clinics due to the extended burden on the health care system, all impact access to abortion.^7^ Additionally, in 2021 the most number of state abortion restrictions were enacted than in any year since Roe v. Wade was decided in 1973, making it the worst year for abortion access.^8^

There has been no discussion on the impact of the pandemic on AAPIs' access to abortion care. We discuss challenges that are specific to AAPIs in accessing sexual and reproductive health (SRH) care, especially abortion, and how these are compounded by the lack of appropriate data and methods. We then discuss the added complexity that comes with accessing abortion care during a pandemic and provide recommendations for research methods to better reach these populations.

## Challenges That AAPIs Face in Accessing Abortion Care

Language barriers and a lack of affordable high-quality interpretation and translation services make access to health care especially burdensome for AAPI communities.^9^ Beyond language, AAPIs face numerous other challenges in accessing health care—discrimination based on culture and religion; lack of culturally appropriate health care; lengthy immigration and citizenship processes that restrict access to social safety nets such as Medicaid; risk of deportation; increased exposure to domestic violence and/or human trafficking; widening wage gaps; and insufficient access to social support systems such as paid sick and family leave.^9^

AAPIs face additional challenges with respect to accessing sexual and reproductive health services—such as viewing sex as taboo, indulging in risky behaviors due to strict cultural expectations and competing social pressures, use of less effective contraception at much higher rates, high birth rates, and differences based on nativity.^9^ In addition, anti-abortion regulations have increased dramatically in middle and southern U.S. states—regions with the fastest growing AAPI populations.^9^ Furthermore, there are bans on sex-selective abortions that have been introduced in some states, which are based on the stereotype that AAPIs might prefer sons over daughters.

Such bans target and discriminate against AAPIs and pose a dangerous risk, as a misunderstanding compounded by linguistic barriers could result in denial of care.^10^ In addition, many states with the fastest growing AAPI populations are among those that severely restrict Medicaid coverage of abortion to the narrowest circumstance.^9^ Nearly one in five AAPI women rely on Medicaid; paying for an abortion out of pocket can be devastating for many low-income AAPIs.^9^ “Fetal rights” laws are also being used to target and criminalize AAPIs.^11,12^ Criminalizing people for pregnancies that do not result in childbirth further restricts the reproductive freedom of AAPIs.

Although AAPIs use contraception at rates similar to other women of color,^13^ they tend to use less effective contraceptive methods at much higher rates than other races and ethnicities.^9^ Additionally, within AAPIs, there exist differences in sexual and reproductive health utilization based on nativity.^14–17^ For instance, foreign-born Asians are less likely to receive SRH-related screenings than their U.S.-born counterparts.^14^ They are also less likely to be users of moderately effective methods of contraception than those who are U.S. born.^15^ The limited data that exist suggest that abortion incidence among AAPIs may be relatively high.^9,17^

A 2008 survey of U.S. women who accessed abortions (procedure type not specified) found that 7% identified themselves as AAPI.^17^ In the same study, of the women seeking abortions who were foreign born, almost a quarter of them were Asian or South Asian. A study disaggregating the abortion rates among Asians in New York City in 2014–2015 found that although the abortion rate among Asian women was lower than that among other major racial/ethnic groups, when disaggregated by country of origin, the abortion rates were very different.^16^

Indian women had the highest abortion rate (30.5 per 1000 women), whereas Korean women had the lowest (5.1 per 1000 women), and the abortion rate for U.S.-born Asian women was ∼1.5 times higher than foreign-born women. The differences in abortion rates could be partly attributed to the differences in abortion access that the groups may have due to variability in accessing health coverage, logistic and financial resources, and supportive social networks.^16^ Additionally, immigrant groups may face additional challenges such as limited English proficiency and sociocultural norms that stigmatize abortion, which may further limit their access to abortion.^16^

Nationally representative studies that collect data on AAPIs tend to aggregate this diverse range of communities into a single category, thereby dismissing important differences by subgroup. Much of the knowledge of AAPI health comes from studies wherein AAPIs have been grouped together or a single subgroup has been examined. Policies that are based on data that aggregate all AAPIs into one single group, or extrapolates a subgroup to a wider population, can gloss over the significant differences among this population and this could lead to dangerous health outcomes.^18,19^ Furthermore, data collected on AAPI people are largely unable to be disaggregated to gain insights into lesbian, gay, bisexual, transgender, queer (LGBTQ), and gender expansive people.^9^

## AAPIs' Access to Abortion During the Pandemic

We hypothesize that AAPIs may face additional challenges and restrictions—at an individual level due to cultural norms and abortion stigma, and at a systemic level due to racial inequities, immigration status, and lack of reproductive justice^*^—in accessing abortion. The pandemic adds further complexity to both abortion access, in general, and AAPIs' ability to access care. Hence, AAPIs are more likely to face additional challenges in accessing abortion care during the pandemic, with AAPI immigrants potentially experiencing further disruptions to care.^21^ Further research is needed to untangle the complex web of intersectional factors that influence AAPIs' access to and their experiences of accessing abortion services during the pandemic.

## The Need for Community-Informed Research to Address Evidence Gaps

Given the diversity among AAPIs, it is challenging (and expensive) to collect data that can take into account differences in country of origin, degrees of cultural orientation and assimilation, ethnic and racial groups, age, gender, class, and sexual orientation, among other factors. This dearth of disaggregated nationally representative data, and a complete lack of nuanced qualitative data at the individual level already results in a very poor understanding of AAPIs across the country and their perspectives on abortion, leading to very little evidence being available to formulate programmatic, clinical, and policy recommendations to increase their access to abortion.

Community-informed research is a potential mechanism to better understand and address critical reproductive health issues within AAPIs. Studies have shown the important role that community-informed research involving community-based organization (CBO) can play. A study of Chinese communities in the United States showed that community–academic partnerships can help build trust with communities, encourage participation, and design culturally and linguistically appropriate instruments to generate evidence that can improve the lives of minorities.^22^ Most community-informed studies of AAPIs thus far have focused on health risks such as heart disease, liver cancer, breast cancer, and cervical cancer^23,24^; however, no studies exist on access to abortion.

To create reproductive health policies and practices that are responsive to the needs of the diverse range of AAPI communities, it is crucial to cocreate this study with AAPIs, so that it best reflects our own needs and priorities. Additionally, existing research demonstrates how culture impacts abortion experience.^25–27^ Research with AAPIs should be firmly rooted in the culture, and cognizant of the extent to which living in the United States may (or may not) influence both foreign- and U.S.-born AAPIs. We need to generate quality, disaggregated, qualitative, and quantitative data that can provide insight into the lived realities of AAPIs, which can result in advocating for policies, and informing program design, that can ultimately lead to increased access to stigma-free equitable abortion access for AAPIs.

## Abbreviations Used

AAPIs: Asian American and Pacific Islanders
CBOs: community-based organizations
SFPRF: Society of Family Planning Research Fund
SRH: sexual and reproductive health

## Notes

^*^
Reproductive justice is the human right to maintain personal bodily autonomy, have children, not have children, and parent the children we have in safe and sustainable communities. Reproductive justice is a human right, is about access, not choice, and is intersectional in nature. To achieve reproductive justice, we must analyze power systems, address intersecting oppressions, center the most marginalized, and join together across issues and identities.^20^
